# Oscillating Reactions Meet Polymers at Interfaces

**DOI:** 10.3390/ma13132957

**Published:** 2020-07-02

**Authors:** Alina Osypova, Matthias Dübner, Guido Panzarasa

**Affiliations:** 1 Innovative Sensor Technology IST AG, Stegrütistrasse 14, 9642 Ebnat-Kappel, Switzerland; alina.osypova@gmail.com; 2 Laboratory for Surface Science and Technology, Department of Materials, ETH Zürich, Vladimir-Prelog-Weg 5, 8093 Zürich, Switzerland; duebnermatthias@gmail.com; 3 Laboratory of Soft and Living Materials, Department of Materials, ETH Zürich, Vladimir-Prelog-Weg 5, 8093 Zürich, Switzerland

**Keywords:** polymer brushes, layer-by-layer, Belousov–Zhabotinsky (BZ) reaction, oscillating reactions, chemical clocks, spatiotemporal patterns, periodic actuation, soft robotics, biomimetic

## Abstract

Chemo-mechanical phenomena, including oscillations and peristaltic motions, are widespread in nature—just think of heartbeats—thanks to the ability of living organisms to convert directly chemical energy into mechanical work. Their imitation with artificial systems is still an open challenge. Chemical clocks and oscillators (such as the popular Belousov–Zhabotinsky (BZ) reaction) are reaction networks characterized by the emergence of peculiar spatiotemporal dynamics. Their application to polymers at interfaces (grafted chains, layer-by-layer assemblies, and polymer brushes) offers great opportunities for developing novel smart biomimetic materials. Despite the wide field of potential applications, limited research has been carried out so far. Here, we aim to showcase the state-of-the-art of this fascinating field of investigation, highlighting the potential for future developments and providing a personal outlook.

## 1. Introduction

The development of novel functional materials through bioinspired or biomimetic approaches is based on the study of natural, biogenic structures and their imitation by artificial materials, usually with the goal of replicating desirable properties [1,2].

Although the natural world provides countless inspiration models for the design and development of novel materials, the synthesis of artificial systems capable of responding to stimuli in a controllable and predictable way still faces significant challenges. In this regard, it is especially difficult to mimic biological systems in which orchestrated responsive behaviors are generated by structural and compositional gradients at various length scales. To tackle these challenges, many stimuli-responsive material systems have been developed, especially polymer-based ones: from solutions to surfaces and interfaces, to gels. The predominance of soft matter for these studies is not surprising, considering its unique properties and especially its sensitivity to physico-chemical stimuli.

**Figure 1:**
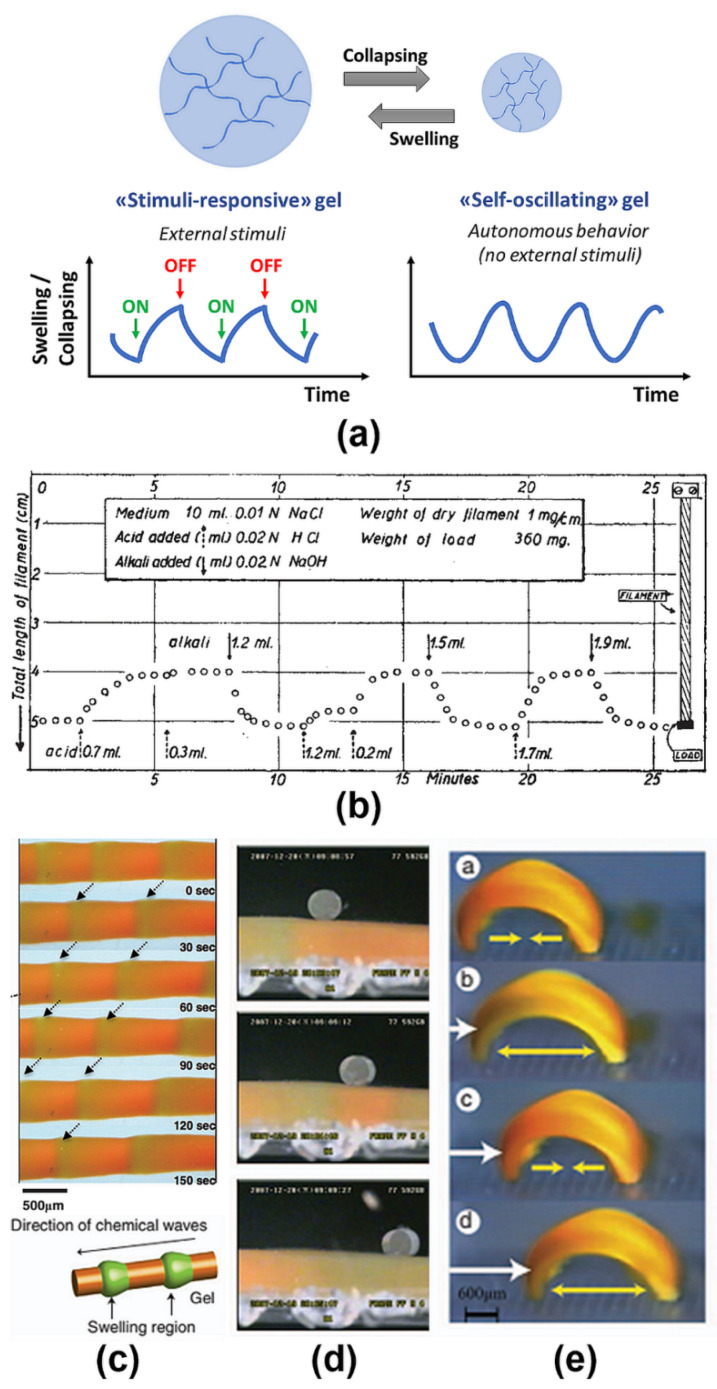
(**a**) Schematic representation of the conceptual difference between conventional, externally controlled gels and self-oscillating autonomous gels. Adapted from [37], Nature Publishing Group, 2014. (**b**) pH-Responsive polymer fibers as artificial muscles: the swelling and collapsing of chains, controlled by the external addition of bases or acids, results in mechanical movement (lowering and lifting of a load, respectively). Reproduced with permission from [7], Nature Publishing Group, 1950. (**c**–**e**) Self-oscillating gels, powered by the Belousov–Zhabotinsky reaction, as chemo-mechanical transducers: (**c**) peristaltic motion, (**d**) mass transport, (**e**) self-walking gel. Adapted and reproduced from [37], Nature Publishing Group, 2014.

Nevertheless, artificial systems based on conventional stimuli-responsive polymers and gels need to be actuated by an externally controlled on-off switching of stimuli. As such, they provide only one action, such as either expanding or collapsing, towards a stable equilibrium state (Figure 1a). In other words, they miss the ability to spontaneously change with temporal periodicity (the so-called ‘temporal structure’). In contrast, many physiological systems can maintain rhythmical oscillations under constant environmental conditions, and act in a dynamic nonequilibrium state—i.e., they are self-regulating—as represented by the autonomic heartbeat [3].

A huge variety of living organisms can directly convert chemical energy to mechanical energy, for example in their muscles, through the hydrolysis of adenosine triphosphate (ATP). This is in stark contrast to man-made thermal engines, which need to convert chemical energy (e.g., from carbon or gasoline) into thermal energy (heat) first and only turn it into mechanical work subsequently. The difference between these two mechanisms is striking, and so is their relative efficiency.

In the 1940s, Katchalsky et al. studied the development of artificial systems able to convert chemical free energy directly into mechanical work [4]. It was reported that chemo-mechanical transduction under isothermal conditions could be obtained as a result of phase transitions in both artificial (e.g., poly(acrylates)) and natural (e.g., collagen [5]) polymers induced by differences in pH [6,7] or salt concentration [4] (Figure 1b). Nevertheless, breakthroughs in the field could be achieved only decades later, when Yoshida and collaborators coupled hydrogels with oscillating reactions [8], giving birth to the development of the fertile field of self-oscillating polymer systems [9,10,11].

Oscillatory mechanical movements in polymer systems can be induced through the application of alternate inputs of external stimuli, whether physical (e.g., light [12], heat [13], electric current [14,15]) or chemical (e.g., pH [16], redox [17]), alone or in combination (e.g., heat and light [18], light and photoreactive molecules [19]). Chemically induced oscillations can be generated both with and without external control—in this latter case by embedding chemo-mechanical feedback mechanisms in the polymer system itself.

Chemo-mechanical feedback is a dynamic, iterative interplay between chemical signals and macromolecular reconfigurations, resulting in motions that connect space and time scales. It forms the core of living organisms’ adaptive abilities and is built into their most fundamental materials. Evoked in synthetic materials, it can give rise to a fascinating diversity of complex multiscale responsive behaviors [20].

The exploitation of oscillating reactions as driving mechanisms for molecular motors and machines enables the generation of systems in which chemical energy is converted directly into mechanical energy. While oscillating, the change in the oxidation state of a certain species and/or release of energy from the reaction itself periodically engages with the ‘mechanical’ part that, in turn, enacts the machine. The response of the machine is potentially tunable to its intended application.

Self-oscillating polymers are fascinating embodiments of De Gennes’ famous quote “a very mild chemical action has induced a drastic change in mechanical properties: a typical feature of soft matter” [21]. The full potential and versatility of stimuli-responsive hydrogels as chemo-mechanical transducers, converting chemical energy into mechanical motion, has been demonstrated in subsequent studies by the group of Yoshida. Prototypical artificial muscles [22], cilia [23], self-walking gels [24], self-beating micropumps for microfluidics [25] and other similar devices have been described based on this working principle. Thanks to the commitment of different researchers, the applicability of this principle has been demonstrated across the scales, from single chains [26,27,28], molecular complexes [29] and micelles [30] to polymersomes [31,32,33], droplets [34] and micro- and macro-gels [35] (Figure 1d).

While self-oscillating gels have been detailed in many excellent reviews [36,37], self-oscillating chains, layer-by-layer assemblies and polymer brushes did not receive any equivalent attention so far, despite their relevance for the development of smart surfaces. Here we want to address this lack of visibility by providing a comprehensive state-of-art of the research on self-oscillating polymers at interfaces.

### 1.1. Stimuli-Responsive Polymers at Interfaces: What’s So Special about Them?

Any material is responsive, since changing its environment always produces modifications of its properties. For this reason, there is no obvious nor universal definition for what should be recognized as “responsive behavior”, and this term mostly refers to materials’ applications. For many applications, a steep and well noticeable change (“switching“) of the given properties is highly desirable, for example: transitions from swollen to collapsed, from wetting to non-wetting, from adhesive to non-adhesive, from attractive to repulsive, and so on. It is reasonable then to define “response” as a sizeable effect, useful for the given application, if it appears as a change of the system’s properties resulting from a change in the surrounding environment. In polymeric systems, such changes of properties are typically (but not necessarily) accompanied by changes of their molecular conformations, which are assigned as responsive properties.

In three-dimensional polymer networks, such as in gels, the individual structural components (chains, crosslinks) are responsible for localized responsiveness. However, to obtain collective and orchestrated responsiveness to external or internal stimuli, certain spatial and energetic network properties are necessary, which do vary significantly between stimuli-responsive polymeric solutions, surfaces and interfaces, and gels. Moving from a solution to surfaces, interfaces and gels, segmental mobilities of polymer chains decreases due to significant spatial restrictions manifested by smaller displacement vectors in the *x*, *y*, and *z* directions. Dimensional responsiveness can be attainable with minimal energy input in systems with a higher solvent content, thanks to a different degree of restrictions on the mobility of polymeric chains. Greater challenges arise when designing gels that need to maintain their mechanical integrity while responding to stimuli. Spatial limitations result from restricted mobility within the network, thus imposing limits on the magnitude of the stimuli-responsive behavior. It is necessary to generate networks capable of inducing small (at the molecular level), yet orchestrated changes that lead to significant physico-chemical responses upon external or internal stimuli [38].

Spatial restrictions also dictate the energetic requirements to undergo transitions from one state to another, while maintaining physical/chemical integrity. For practical applications it is desirable to maintain the state at the equilibrium energy to preserve the functionality of the network and, at the same time, generate an architecture that will require significantly lesser amounts of energy to exhibit stimuli-responsiveness. The latter can be represented by two (usually smaller) metastable energy minima. Transitions between these minima represent the energy required for a system to go from one state to another. Examples of such transitions are conformational changes (e.g., molecular cis-trans interconversions), rearrangements induced by hydrogen bonding, protonation–deprotonation, order-disorder transitions, or aggregation-dissociation phenomena. These lower energy transitions may or may not be reversible, and their energy requirements depend on initial physical and chemical states [38].

In polymers at interfaces, due to the anchoring of one end of the polymeric segment to the surface, the restricted freedom of movement is “transmitted” along the chain. Constraints due to the end grafting of the polymer chains introduce a different response compared to that of isolated chains in solution and of chains in a crosslinked network. The chains stretch out of the grafting surface until the excluded volume effect is compensated by elastic energy (stretching entropy) of polymer coils [39]. The energy required to respond is, for the segments further away from the anchoring point, a function of the distance from the surface. The more distant the segments are to the surface-anchoring points, the less energy input is required to actuate transitions because more space and free volume are available. Thanks to the protagonist role of surface forces, leading to the emergence of peculiar behaviors—such as enhanced stimuli-responsiveness—polymers at interfaces offer unique possibilities compared to bulk polymeric materials.

Polymers at interfaces can be classified in three main categories—single chains, layer-by-layer (LbL) assemblies and polymer brushes—using two basic criteria: (i) the interaction between the chains and the interface (adsorption *vs*. grafting), and (ii) the surface density or coverage. Single polymer chains represent the lowest level of surface coverage, while polymer brushes the highest. Here, the discussion of different kinds of self-oscillating polymers at interfaces will thus follow this order.

### 1.2. Chemical Clocks and Oscillating Reactions

Clock reactions are chemical systems in which a delay of well-defined length takes place after mixing of the initial reagents and is followed by the prompt appearance of the reaction product(s) [40]. The first reaction of this kind (an “iodine clock”) has been described by Landolt in 1887 [41], based on the reaction between iodate and sulfite in acidic conditions. Later, in 1929, Wagner demonstrated the sudden acid-to-alkali pH change in the reaction between formaldehyde, sulfite and bisulfite, what became known as the “formaldehyde clock” [42,43,44,45]. Notable exceptions to the previously mentioned controlled and reproducible behavior are the so-called “crazy clocks”, such as the chlorite-iodide (CI) reaction [46,47], whose high sensitivity to initial reaction conditions, including stirring, makes their lagtime stochastic. Among such chemical clocks, the so-called chemical oscillators are probably the most fascinating ones. Compared to clock reactions, chemical oscillators switch contiounsly and autonomously in between two or more “states” (e.g., in pH and/or redox potential), making them especially attractive for the development of chemo-mechanical actuators.

In 1921 Bray demonstrated that the catalytic decomposition of hydrogen peroxide by iodate occurs with a periodic behavior, the first example of a homogenous oscillating chemical reaction [48]. Almost fourty years later, Belousov observed periodic color changes during the cerium-catalyzed oxidation of citric acid by bromate, and optimized the conditions necessary to obtain stable oscillations. These results were first published in 1959 [49]. More than ten years after its original discovery, Zhabotinsky started a detailed investigation of the Belousov reaction, substituting citric acid with malonic acid and using various metal catalysts [50,51]. Nevertheless, the exact mechanism remained unknown for a long time. It was only in 1972, when Field, Körös and Noyes proposed a skeleton mechanism, which was named FKN after their initials, for this complex reaction [52]. According to the FKN mechanism, the Belousov–Zhabotinsky (BZ) reaction can be divided into three main chemical sub-systems, linked together by feedback relations: (i) the concomitant reduction of bromate $BrO_{3}^{-}$ and scavenging of the inhibitor bromide $Br^{-}$ (Equations (1)–(3)), (ii) an autocatalytic reaction of bromous acid $HBrO_{2}$ coupled with the oxidation of the metal catalyst (e.g., a ruthenium-bipyridyl Ru-bpy complex) (Equation (4)), and (iii) the reduction of the metal catalyst by reaction with the organic substrate (e.g., malonic acid, bromomalonic acid) (Equation (5)).

(1) $$BrO_{3}^{-}+2Br^{-}+3H^{+}\to 3HOBr$$

(2) $$BrO_{3}^{-}+Br^{-}+2H^{+}\to HBrO_{2}+HOBr$$

(3) $$HBrO_{2}+Br^{-}+H^{+}\to 2HOBr$$

(4) $$BrO_{3}^{-}+HBrO_{2}+2Ru{\left(bpy\right)}_{3}^{2+}+3H^{+}\to 2HBrO_{2}+2Ru{\left(bpy\right)}_{3}^{3+}+H_{2}O$$

(5) $$2Ru{\left(bpy\right)}_{3}^{3+}+CH_{2}{\left(COOH\right)}_{2}+CHBr{\left(COOH\right)}_{2}\to Br^{-}+2Ru{\left(bpy\right)}_{3}^{2+}+other\;products$$

The core of the oscillating process is the autocatalytic formation of bromous acid $HBrO_{2}$ (positive feedback), which is inhibited by the later formation of bromide (negative feedback). The metal ions of the catalyst are oxidized and reduced stoichiometrically during each cycle, leading to periodic color and/or fluorescence variations, depending on the properties of the catalyst chosen. The catalytic amount of metal catalyst limits the autocatalytic formation of the intermediate and maintains oscillations in a closed reactor. The Belousov–Zhabotinsky (BZ) reaction is the most used and best studied homogeneous bulk oscillating chemical reaction to perform experiments of any type. Over time, other homogeneous oscillating reactions have been proposed, such as the Briggs–Rauscher (BR) [53] or the chlorite-iodate-X (where “X” is a substrate, such as arsenite) [54]. Such long-lasting autonomous oscillations are rare, and most classical chemical oscillators are usually operated in an open continuously stirred tank reactor (CSTR) fed constantly with fresh reactants to sustain oscillations.

Certain clock reactions can be transformed into oscillating systems by operating in flow conditions. For example, the bromate–sulfite (BS) reaction is an acid-autocatalyzed chemical clock [55]; when ferrocyanide is introduced, and the reaction is performed under flow, the resulting bromate–sulfite–ferrocyanide (BSF) system can produce periodic pH changes, usually between pH 6 and pH 3 [56]. The actual mechanism is complex, but it involves two main processes: (i) the oxidation of sulfite $SO_{3}^{2-}$ by bromate (Equations (6) and (7)), and (ii) the oxidation of ferrocyanide by bromate (Equation (8)):

(6) $$SO_{3}^{2-}+H^{+}⇌HSO_{3}^{-}$$

(7) $$BrO_{3}^{-}+3HSO_{3}^{-}\to Br^{-}+3SO_{4}^{2-}+3H^{+}$$

(8) $$BrO_{3}^{-}+6Fe{\left(CN\right)}_{6}^{4-}+6H^{+}\to Br^{-}+6Fe{\left(CN\right)}_{6}^{3-}+3H_{2}O$$

The first process transforms a weak acid (bisulfite $HSO_{3}^{-}$) in a strong, fully ionized acid (hydrogen sulfate $HSO_{4}^{-}$) generating hydrogen ions (positive feedback), while the second consumes them (negative feedback): as a result, pH oscillations develop in the system.

Chemical clocks and oscillating reactions can be coupled to polymer systems in two ways: (i) the chemical clock is used as an in situ stimuli (pH, redox potential) generator to actuate a stimuli-responsive polymer, or (ii) the polymer itself is embedded with functional elements for the clock/oscillating reaction. For both kinds of design, however, the available elements are still limited.

## 2. Chemo-Mechanical Oscillations in Surface-Grafted Single Polymer Chains and Layer-by-Layer Assemblies

### 2.1. Self-Oscillating Surface-Grafted Single Polymer Chains

**Figure 2:**
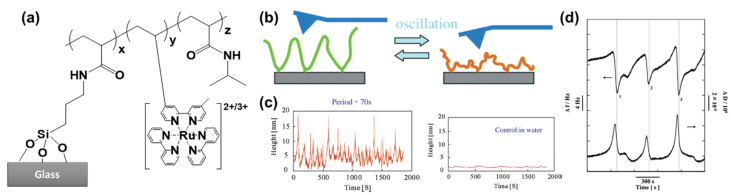
Self-oscillations in surface-grafted polymer chains. (**a**) Structure of the grafted polymer. (**b**) Schematic representation and experimental results for an oscillating chain probed by (**c**) atomic force microscopy (AFM) and (**d**) quartz-crystal microbalance with dissipation (QCM-D). Adapted and reproduced with permission from [57,59], American Chemical Society, 2006 and 2013. Conditions for the BZ reaction: [HNO_3_] 0.3 M, [NaBrO_3_] = 0.3 M, [malonic acid] 0.1 M.

Mechanical oscillations induced by an oscillating reaction in a system of surface-grafted polymer chains were first described in 2006 by Ito et al. [57]. This work appeared ten years after a seminal work on self-oscillating gels [8], and four years after a study on self-oscillating free polymer chains in solution [58], describing a thermoresponsive N-isopropyl acrylamide polymer, modified with pendant ruthenium tris(2,2′-bipyridine) (Ru(bpy)_3_) and N-succinimidyl (NAS) groups, from now on referred to as poly(NIPAAm-*co*-Ru(bpy)_3_-*co*-NAS). The co-polymer was synthesized by free radical polymerization and covalently immobilized on aminosilane-modified glass substrates through the NAS groups (Figure 2a). The ruthenium complex acted as a catalyst for the BZ reaction, which took place when the polymer-grafted substrates were immersed in a reaction medium containing proper concentrations of malonic acid, sodium bromate and nitric acid. Changes in the redox state of the metal catalyst reflected in changes of the lower critical solution temperature of the polymer chains, and consequently in their periodic swelling and collapsing. Thus, in this kind of configuration, the polymer plays an active role in generating the oscillations.

The self-oscillations of the polymer chains were observed by atomic force microscopy (AFM). The amplitude of the oscillations reached 15 nm with a periodicity of 70 s (Figure 2b,c). Some irregular, chaotic behavior was observed, presumably due to the absence of homogeneous stirring, a condition which is known to influence the evolution of the BZ reaction in solution. No oscillations were detected in the control experiments, i.e., when the polymer-modified substrates were immersed in pure water (Figure 2c).

In a subsequent work, Hara et al. [59], immobilized poly(NIPAAm-*co*-Ru(bpy)_3_-*co*-NAS) on aminothiol-modified, gold-coated quartz resonators. The periodic swelling and collapsing of the chains, induced and controlled by the BZ reaction, was measured in terms of changes in the resonance frequency Δf and dissipation ΔD by means of quartz-crystal microbalance with dissipation (QCM-D) (Figure 2d).

### 2.2. Self-Oscillating Layer-by-Layer Assemblies

Layer-by-layer (LbL) deposition is a versatile thin film fabrication technique, first described by Decher et al. in 1991 [60,61]. Films are formed by depositing alternating layers of materials, usually polymers, with washing steps in between. The layered polymer structures obtained with this technique have been used for many different applications, from sensors to smart surfaces [62,63,64,65].

**Figure 3:**
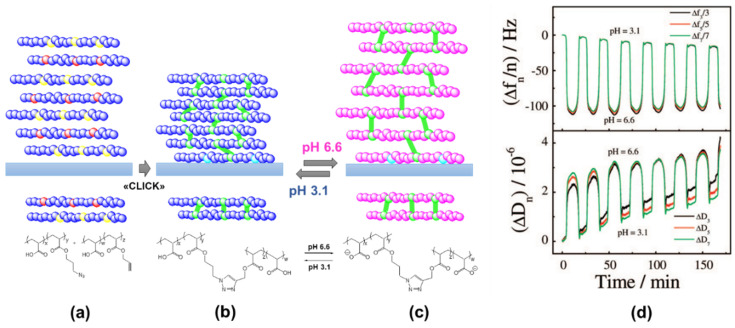
(**a**–**c**) Chemical structure and schematic representation of the pH-oscillating layer-by-layer assembly, crosslinked with a click chemistry approach. (**d**) Corresponding frequency and dissipation shifts monitored by QCM-D. Reproduced with permission from [66], American Chemical Society, 2008.

In 2008, Tang et al. reported the oscillatory behavior of a pH-responsive LbL assembly, which was studied by means of QCM-D [66]. The multilayer was assembled by the assembly and “click” reaction of poly(acrylic acid-*co*-3-azidopropyl acrylate) (poly(AA-*co*-Az)) and poly(acrylic acid-*co*-propargyl acrylate) (poly(AA-*co*-PA)) directly on the QCM crystal resonator, which was previously functionalized with a primer layer of alkyne-modified branched poly(ethyleneimine) (Figure 3a). The crosslinking points guaranteed the mechanical stability of the assembly during the oscillations, preventing its detachment (Figure 3b,c).

The multilayer was then subjected to a continuous flow of the bromate–sulfite–ferrocyanide (BSF) reaction mixture. As discussed before, by constantly feeding fresh reactants, the BSF reaction can be forced to oscillate in the pH range 3.1–6.6. Given the pK_a_ ∼4.5 of the carboxylic groups in the polymeric multilayer, periodic swelling and shrinking were observed with response times of about 90 s and 25 s, respectively. These changes reflected in the behavior of Δf and ΔD, as shown by QCM-D (Figure 3d). Both Δf and ΔD exhibited periodic changes in accordance with the pH oscillations of the BSF reaction. The decrease of Δf corresponding with an increasing pH from 3.1 to 6.6 reflected the swelling of the multilayer due to electrostatic repulsion, while the oscillation of ΔD reflected the multilayer change between a compact (pH 3.1) and loose (pH 6.6) structure (a swollen multilayer is more viscous and can damp the shear wave more efficiently than a collapsed one). 

Compared to the previously discussed BZ reaction-powered system, in this configuration the polymeric structure plays a passive role, as it only responds to the oscillations induced by the reaction network.

## 3. Self-Oscillating Polymer Brushes

Polymer chains which are attached by one end to a surface, when a high enough density coverage is achieved, stretch away from the substrate to reduce their excluded-volume interactions. Such structures are called “polymer brushes” [67,68] and have been the subject of intensive theoretical studies for decades, before polymerization techniques became refined enough to allow their thorough experimental exploration. If the chain growth proceeds directly from the substrate, which is what happens when initiators are grafted on a surface, the approach is called “grafting-from” to distinguish it from “grafting-to” (in which pre-formed chains are adsorbed or covalently bound to a surface). Design possibilities have been enriched by the introduction of controlled radical polymerization techniques, such as nitroxide-mediated radical polymerization (NMP), reversible addition fragmentation chain transfer (RAFT) and atom transfer radical polymerization (ATRP) [69,70]. Polymer brushes have since then been exploited for developing functional surfaces with tunable properties in response to external stimuli [71,72,73,74,75,76,77,78,79,80,81].

**Figure 4:**
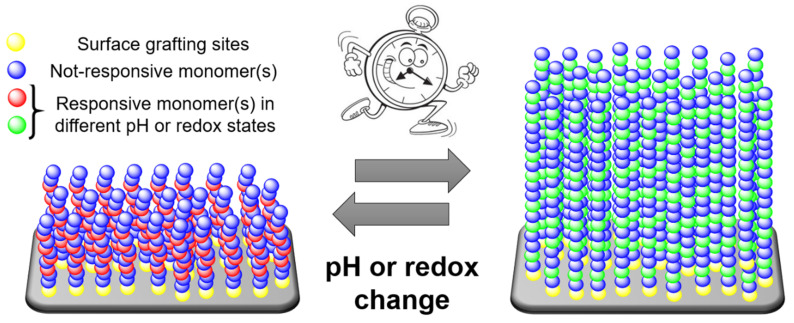
Schematic representation of self-oscillating polymer brushes, periodically swelling and collapsing in a closed homogeneous environment without the need for external stimuli, an autonomous behavior of great interest for biomimetic applications.

Decorating surfaces or interfaces with arrays of polymer chains is an effective strategy to manipulate their properties, introducing new features and functions. Living organisms make widespread use of this principle whenever “smart” surfaces are needed. Excellent examples are the glycocalyx, also known as the pericellular matrix, that surrounds different cell membranes [82], and the mucin-based lubricating layer between bones, which have inspired the development of cartilage-mimicking artificial polymer brushes [83]. In the framework of smart surfaces, self-oscillating polymer brushes (Figure 4) could offer great advantages in terms of, e.g., mass transport and controlled gating, explaining the interest about their development and optimization.

**Figure 5:**
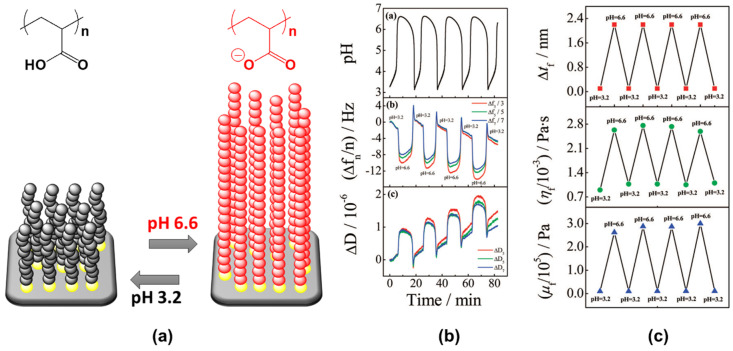
(**a**) Schematic representation of pH-responsive poly(acrylic acid) brushes. (**b**) The observed shifts in frequency and dissipation caused by pH oscillations. (**c**) Deducted changes in brush thickness, shear viscosity and elastic shear modulus. Reproduced with permission from [84], American Chemical Society, 2008.

The first report on self-oscillating polymer brushes was published in 2008 by Liu and Zhang [84]. Poly(acrylic acid) brushes, obtained by surface-initiated (SI) ATRP (Figure 5a), were shown to exhibit periodic conformational changes when exposed to a continuous flow of the bromate–sulfite–ferrocyanide (BSF) reaction mixture. The changes in frequency and dissipation, measured with QCM-D, indicated the periodic swelling and collapse of the brushes because of the oscillations in pH. The changes in thickness, viscosity, and elastic modulus suggested that an oscillation of the total interface took place (Figure 5b,c). In this system the polymer chains are simply actuated from the pH stimuli received from the environment, and do not actively participate to the oscillations.

**Figure 6:**
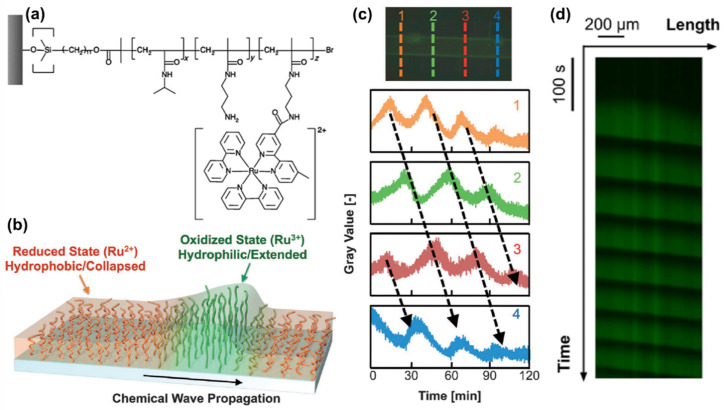
(**a**) Structure of the grafted-from self-oscillating polymer brushes (SOPB). (**b**) Schematic representation of the propagation of a chemical wave in the SOPB. (**c**) Top: fluorescence microscopy images of a glass capillary modified with the SOPB; bottom: oscillating profile of fluorescence intensity for each SOPB position. Adapted and reproduced with permission from [85], Wiley, 2013. (**d**) Spatiotemporal pattern of chemical wave propagation in a SOPB (adapted and reproduced with permission from [86], American Chemical Society, 2015). In both (**c**,**d**) the Belousov–Zhabotinsky (BZ) reaction conditions were: [HNO_3_] 0.81 M, [NaBrO_3_] = 0.15 M, [malonic acid] 0.1 M.

In 2013, a self-oscillating polymer brush based on the BZ reaction was reported [85], based on the design of grafted self-oscillating single chains. A polymer brush made of a poly(NIPAAm-*co*-NAPMAm-*co*-[Ru(bpy)_3_]NAPMAm) random copolymer (Figure 6a) was fabricated with SI-ATRP, and its active behavior (Figure 6b) was demonstrated as oscillating profiles of fluorescence intensity (Figure 6c), thanks to the redox-state dependent fluorescent properties of the Ru-bpy complex. In 2015, these self-oscillating polymer brushes have been shown to be able to generate autonomous propagation of the excited pulse of the oxidized area (i.e., with hydrophilic, extended, swollen polymer chains) due to a reaction–diffusion mechanism (a “chemical wave”) [86]. The observed period was shorter than that typically observed in gel systems, since the thickness of the polymer brush layer (30–100 nm) was much smaller than the size of bulk gels (100–1000 μm). Additionally, since the BZ substrates were homogeneously distributed in the reaction medium, the propagation of the chemical waves did not occur in a single, well-defined direction but rather in a random manner (Figure 6d). Furthermore, this study clarified the importance of a proper selection not only of the BZ reaction conditions, but also of the concentration of ruthenium-based catalyst immobilized into the polymer brushes, to induce stable oscillations.

**Figure 7:**
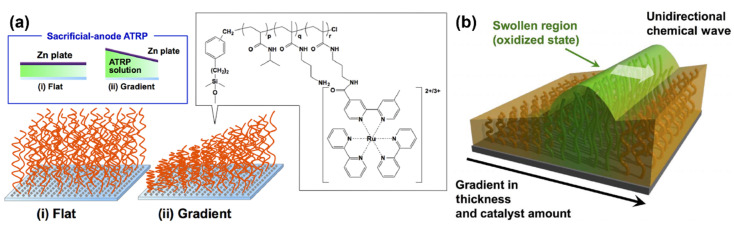
(**a**) Preparation of flat and gradient self-oscillating polymer brushes by sacrificial anode ATRP. The chemical structure of the oscillating polymer is shown in the inset. (**b**) Illustration of the self-oscillating gradient polymer brushes showing the unidirectional propagation of chemical waves generated by the Belousov–Zhabotinski reaction. Adapted and reproduced from [87], American Association for the Advancement of Science, 2016.

Masuda et al. [87] addressed this challenge in 2016, by fabricating a gradient of poly(NIPAAm-*co*-NAPMAm-*co*-[Ru(bpy)_3_]NAPMAm) brushes using a sacrificial anode-mediated SI-ATRP approach [88]. The resulting system, described as an artificial model for ciliar movement, exhibited autonomous wave propagation through polymer chains at the nanometer scale (Figure 7). In that case, the direction of the chemical waves was determined by a gradient in the thickness of the polymer brush layer and, consequently, in the amount of ruthenium-based catalyst. The gradient polymer brush induced a unidirectional propagation of the chemical wave from the region with low metal catalyst amounts to the region rich in metal catalyst. Nevertheless, the direction of wave propagation on the polymer brush surface was random. Although chemical waves were controlled along a 1D path on such graded self-oscillating polymer brush surfaces, the direction control was active only over a limited distance in 1D. In view of potential applications of self-oscillating polymer brushes, controlling the direction of the propagating chemical wave is an important factor.

**Figure 8:**
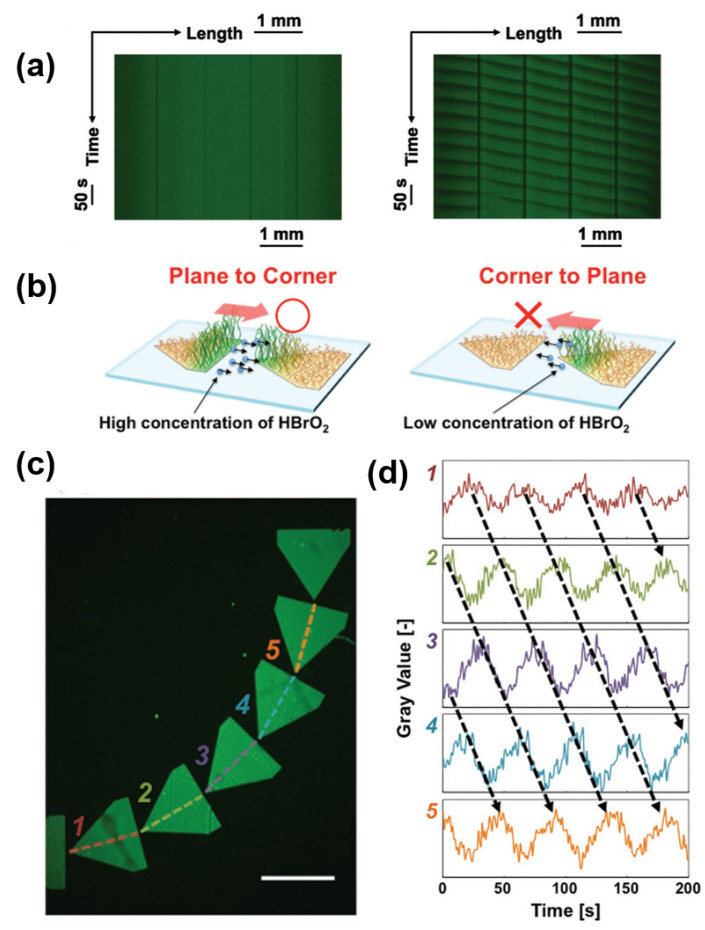
(**a**) Development of spatiotemporal patterns in ordered brushes arrays, showed by lining up one-line images along the dotted line on pentagonal arrays with gap distances of 0 µm (left) and 50 µm (right). The dark areas inside each pentagon patterns represent the oxidized state of the Ru-bpy catalyst. (**b**) Schematic representation of the selective plane-to-corner diffusion of the chemical information vector HBrO_2_, explaining the regular propagation in space of the oscillations. (**c**) Fluorescence image of a curved array of self-oscillating polymer brushes pentagons, and (**d**) oscillating profile of fluorescence intensity at each pentagonal pattern. Scale bars are 1 mm. Adapted and reproduced with permission from [89], Wiley, 2017.

Homma et al. [89] applied patterning as an alternative method to induce controlled nanoactuation in a self-oscillating polymer brush, to increase the control over the direction of the oscillation (Figure 8). It is known from the seminal work of Agladze et al. [90] that patterning of reactive and non-reactive areas with appropriate shapes can be an effective way to control the propagation of chemical waves. The self-oscillating polymer brushes have been shown to generate autonomous propagation of the excited pulse of the oxidized (i.e., hydrophilic, extended polymer chains) area due to a reaction-diffusion mechanism (chemical wave). Hypobromous acid HBrO_2_ is proposed as the chemical “information vector”, the diffusion of which is responsible for the generation of the oscillations. This interpretation is in line with the current understanding of BZ in confined media, e.g., in droplets and microemulsions [91].

**Figure 9:**
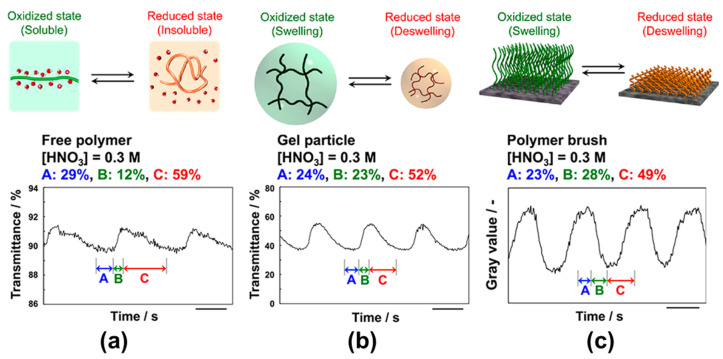
Differences in the oscillation waveforms observed for (**a**) free polymer chains, (**b**) gel particles, and (**c**) polymer brushes. The concentrations of the BZ reaction substrates were as follows: [HNO_3_] = 0.3 M, [NaBrO_3_] = 150 mM, and [malonic acid] = 100 mM. The scale bar is 50 s in all plots. Adapted and reproduced with permission from [92]. American Chemical Society, 2018.

That the mobility of polymer chains could affect the oscillating behavior of self-oscillating polymer brushes was envisioned already in the previously discussed 2015 [86] and 2016 [87] studies, but a dedicated study on the effect of metal catalyst confinement inside the crowded environment of a polymer brush appeared only in 2018. In this work, Masuda et al. [92] compared the self-oscillating behavior of poly(NIPAAm-*co*-NAPMAm-*co*-[Ru(bpy)_3_]NAPMAm) in the form of free chains, gel particles and surface-attached brushes (Figure 9), confirming that in polymer brushes peculiar reaction-diffusion patterns can arise due to chain crowding and reduced accessibility of metal catalyst.

**Figure 10:**
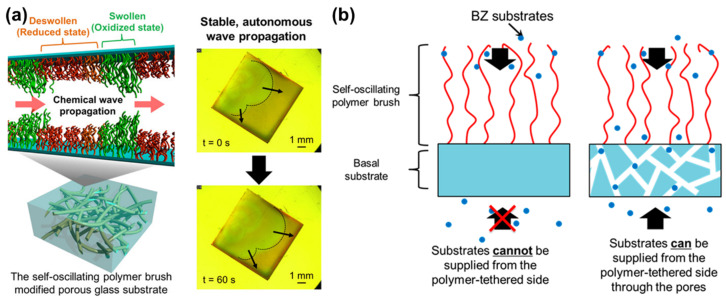
(**a**) Schematic representation (left) and experimental observation (right) of self-oscillating polymer brushes grown from porous glass. (**b**) Illustration of the different supply mechanism of BZ substrates to polymer brushes. In the case of a non-porous substrate, the BZ substrates can only be supplied from the free-end of the polymer brush, while with a porous substrate they can be supplied efficiently both from the free-end and the tethered-end. Adapted and reproduced with permission from [93], American Chemical Society, 2019.

Another important step further for the understanding of self-oscillating polymer brushes has been recently reported by Homma et al. [93]. In this study, poly(NIPAAm-*co*-NAPMAm-*co*-[Ru(bpy)_3_]NAPMAm) brushes were grown on a porous, instead of a solid, substrate (Figure 10). Increasing the surface area would have: (i) improved the metal catalyst immobilization in the brushes, (ii) ensured a more effective supply of the BZ substrates, and (iii) reduced the diffusion of intermediate products (in particular of the chemical information vector HBrO_2_) from the polymer brushes, eventually resulting in more stable oscillations. These hypotheses were confirmed by the experimental results: the wave velocity and diffusion coefficient observed for brushes grafted from porous glass were significantly lower compared to those grown on more conventional substrates, suggesting that a markedly different reaction-diffusion behavior was at play.

## 4. Conclusions and Outlook 

Chemo-mechanical energy conversion can be achieved by coupling an oscillating reaction medium (i.e., the fuel, chemical input) with the conformational changes of polymer chains (i.e., the engine, mechanical output) that act as transducers. The dynamic properties of these systems, summarized in Table 1, have been studied with different techniques, including AFM, QCM-D and fluorescence microscopy. Achieving full control over the oscillating behavior requires to finely tune several physicochemical parameters, including: the amount and spatial distribution of catalyst, the rate constant of the autocatalytic reaction, the diffusion constant of the activator and the activation energies for both reaction and diffusion processes. Much work is still needed to widen the palette of available oscillating systems.

The modification of surfaces with special “clock-polymers”, able to autonomously switch their properties, can lead to breakthroughs for the development of biomimetic autonomous soft interfaces. Applications can be envisioned to control mass of fluid transport in nano- and micro-engineered systems (thanks to peristaltic motion), as well as for developing cilia-like actuators, devices for the periodical release of molecules or ions, for controlling the accessibility of active sites in sensors, and for soft robotics.

However, much research remains to be done before all these ideas could find practical realization. Researchers need to take advantage of the rich palette of architectures, both chemical and topological, made available by recent progresses in surface-initiated polymerization. One important limitation of state-of-art systems is the necessity for relatively harsh reaction conditions, such as low pH and strong oxidizing (or reducing) agents, factors that not only discourage their use for biological-related applications but could also lead to premature degradation and failure of the polymer systems. This latter point is of special relevance for the application of chemical clocks to stimuli-responsive biopolymers, such as cellulose [94] and chitosan [43].

Clock reactions have several advantages compared to oscillating reactions, and recent developments in programmable pH spikes [55,95] and cycles [44] offer a fertile ground for investigation. Moreover, it could be highly rewarding to focus attention on chemical stimuli other than pH and/or redox: complexation-driven supramolecular interactions—e.g., those between iodine and polymers such as poly(vinyl alcohol) (artificial) or starch (natural)—are highly promising in this regard [45]. There exists a huge variety of iodine-based chemical clocks (“iodine clocks”), and preliminary work in this direction has already proven successful [46,96].

## Figures and Tables

**Table 1:** Synopsis of the self-oscillating polymers at interfaces discussed in the present review.

Self-Oscillating Polymer System	Role of the Polymer	Oscillating Reaction	Features	Ref.
BZ-powered surface-grafted chains 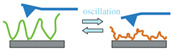	Active	Belousov–Zhabotinsky (BZ)	Chains of random NIPAM-based copolymer functionalized with a Ru-bpy catalyst.	[57,59]
Surface-grafted pH-responsive LbL 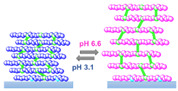	Passive	Bromate–sulfite–ferricyanide (BSF)	Layer-by-layer assembly of acrylic acid-based copolymers crosslinked by click chemistry to avoid layer separation during pH-induced oscillations (swelling-collapsing).	[66]
pH-responsive polymer brushes 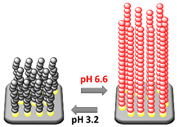	Passive	Bromate–sulfite–ferricyanide	Homopolymer brushes of pol(acrylic acid) prepared by grafting-from with ATRP.	[84]
BZ-powered polymer brushes 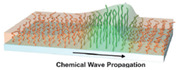	Active	Belousov–Zhabotinsky	Polymer brushes based on NIPAM and incorporating a Ru-bpy catalyst, made by grafting-from on solid surface. Chemical waves observed, propagating randomly.	[85,86]
BZ-powered polymer brushes 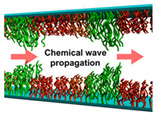	Active	Belousov–Zhabotinsky	Polymer brushes (see above) grown on porous surface. Slower, random propagation of chemical waves thanks to increased diffusivity of chemical species.	[93]
BZ-powered polymer brushes 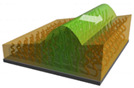	Active	Belousov–Zhabotinsky	Polymer brushes (see above) grown on a solid surface with a thickness gradient. The propagation of chemical waves is controlled from thinner to thicker regions.	[87]
BZ-powered polymer brushes 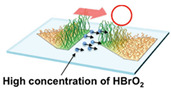	Active	Belousov–Zhabotinsky	Patterned polymer brushes (see above) grown on a solid surface. Controlled unidirectional (plane-to-corner) propagation of chemical waves thanks to preferential diffusion of HBrO_2_.	[89]
